# Gait Analysis in Parkinson’s Disease: An Overview of the Most Accurate Markers for Diagnosis and Symptoms Monitoring

**DOI:** 10.3390/s20123529

**Published:** 2020-06-22

**Authors:** Lazzaro di Biase, Alessandro Di Santo, Maria Letizia Caminiti, Alfredo De Liso, Syed Ahmar Shah, Lorenzo Ricci, Vincenzo Di Lazzaro

**Affiliations:** 1 Unit of Neurology, Neurophysiology, Neurobiology, Department of Medicine, Università Campus Bio-Medico di Roma, Via Álvaro del Portillo 21, 00128 Rome, Italy; a.disanto@unicampus.it (A.D.S.); m.caminiti@unicampus.it (M.L.C.); a.deliso@unicampus.it (A.D.L.); lorenzo.ricci@unicampus.it (L.R.); v.dilazzaro@unicampus.it (V.D.L.); 2 Usher Institute, Edinburgh Medical School: Molecular, Genetic and Population Health Sciences, The University of Edinburgh, EH16 4UX Edinburgh, UK; ahmar.shah@ed.ac.uk

**Keywords:** Parkinson’s disease, gait analysis, diagnosis, symptoms monitoring, wearable, home-monitoring, machine learning

## Abstract

The aim of this review is to summarize that most relevant technologies used to evaluate gait features and the associated algorithms that have shown promise to aid diagnosis and symptom monitoring in Parkinson’s disease (PD) patients. We searched PubMed for studies published between 1 January 2005, and 30 August 2019 on gait analysis in PD. We selected studies that have either used technologies to distinguish PD patients from healthy subjects or stratified PD patients according to motor status or disease stages. Only those studies that reported at least 80% sensitivity and specificity were included. Gait analysis algorithms used for diagnosis showed a balanced accuracy range of 83.5–100%, sensitivity of 83.3–100% and specificity of 82–100%. For motor status discrimination the gait analysis algorithms showed a balanced accuracy range of 90.8–100%, sensitivity of 92.5–100% and specificity of 88–100%. Despite a large number of studies on the topic of objective gait analysis in PD, only a limited number of studies reported algorithms that were accurate enough deemed to be useful for diagnosis and symptoms monitoring. In addition, none of the reported algorithms and technologies has been validated in large scale, independent studies.

## 1. Introduction

Parkinson’s’ disease (PD) gold standard for diagnosis and symptoms monitoring is based on clinical evaluation, which includes several subjective components. The lack of objective and quantitative biomarkers for diagnosis and symptoms monitoring leads to significant direct and indirect healthcare cost. Based on the current diagnostic criteria [1], the diagnostic error rate is around 20% [2]. In addition, PD is a dynamic disease (i.e., symptoms changes during the disease course) that requires continuous adjustment of therapy.

In the early stages of PD, the most effective treatment to alleviate motor symptoms is oral L-DOPA [3]. However, during moderate and advanced stages, in addition to cardinal motor symptoms, the patient may show motor fluctuations and dyskinesia. During this stage, the brain becomes very sensitive to dopamine level fluctuations, and a continuous stimulation (instead of pulsatile drugs administration) may help in controlling motor fluctuations, dyskinesias and cardinal motor symptoms. This stimulation may be pharmacological with levodopa [4,5,6] or dopamine agonists [7], or provided by DBS (deep brain stimulation) [8,9,10,11,12]. During moderate and advanced stages, gait problems, like freezing of gait and reduced balance and postural control, become more evident and unlike cardinal motor symptoms, PD patients respond less to conventional therapy (i.e., oral L-DOPA).

In line with cardinal motor symptoms, to date, gait problems are evaluated with semiquantitative rating scales like the unified Parkinson’s disease rating scale (UPDRS) [13] or the movement disorders society unified Parkinson’s disease rating scale (MDS-UPDRS) [14]. In an effort to improve PD management and move towards a quantitative and home-oriented assessment and recognition of PD motor symptoms, different technologies have been used to evaluate bradykinesia [15,16,17], rigidity [17,18,19,20], tremor [21,22,23] and axial symptoms [24,25,26,27].

Gait impairment is an evolving condition and different patterns of gait disturbances can be detected throughout the progression of the disease [28]: reduced amplitude of arm swing, reduced smoothness of locomotion, increased interlimb asymmetry [29], low speed, reduced step length [29], shuffling steps, increased double-limb support, increased cadence [28], defragmentation of turns (i.e., turning en block), problems with gait initiation [30], freezing of gait and reduced balance and postural control [28].

Some gait features in PD are specific, and get worse during the disease course. An objective and quantitative gait analysis system could, therefore, potentially improve the current practice (semiquantitative gait evaluation) that may aid in diagnosis, symptom monitoring, therapy management, rehabilitation and fall risk assessment and prevention in Parkinson’s disease patients. Among all these promising applications of gait analysis in Parkinson’s disease, we have confined the scope of our review to two main unmet needs in this disorder: the diagnostic error, and the lack of objective biomarkers for motor status discrimination. Therefore, the main aim of this overview is to summarize the most important technologies used to evaluate gait features and the associated algorithms that have shown promise in using gait analysis to aid diagnosis and symptom monitoring in Parkinson’s disease (PD) patients. The scope of the review was confined to studies that showed any promise of being clinically useful (i.e., are both highly sensitive and specific defined as those with at least 80% sensitivity and 80% specificity) for diagnosis or motor status discrimination.

### 1.1. Gait Features

Human gait is a sequence of involuntary movements, cyclically repeated and triggered by voluntary movement. Several components could be used to objectively measure and analyze gait cycle. These components are typically categorized into spatiotemporal, kinematics and kinetics features [31,32].

### 1.2. Spatiotemporal Features

Several spatiotemporal features can be used for gait analysis (Table 1). These features are the more commonly used types of features to objectively describe the gait pattern in healthy subjects and patients with several diseases. Spatiotemporal features could refer to the global gait cycle or to the stride cycle. 

**Figure 1:**
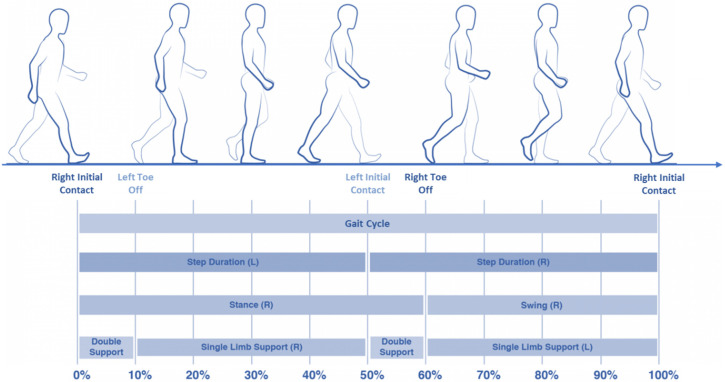
Human gait cycle.

Each gait cycle starts with the initial contact of one foot and ends with a new initial contact of the same foot (Figure 1). One single cycle is composed of a stance and a swing phase: the stance phase is the period during which the foot is in contact with a support surface, and the swing phase is the period during which the same foot is airborne in preparation for the next gait cycle. During the gait cycle, the legs can be individually or simultaneously placed on the ground, so it is possible to identify a single limb support stage, that is the phase during which only one foot is on the surface, and a double limbs support stage during which both legs are on the support surface during a one step cycle (Table 1).

**Figure 2:**
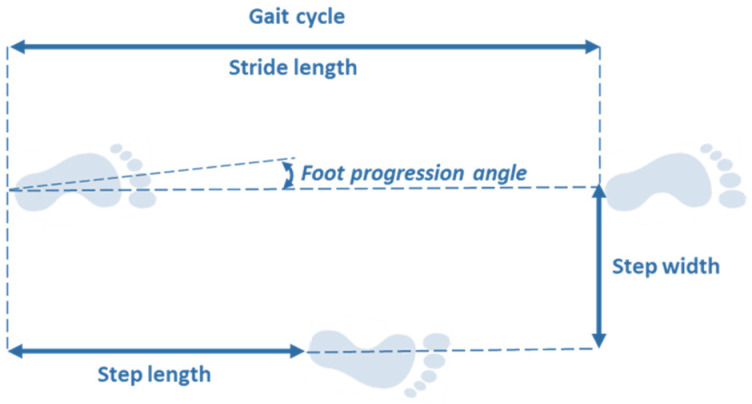
Stride analysis of a single gait cycle.

The step and the stride are defined as the length/duration between 2 successive events of same type on opposite limbs, and on the same limb, respectively (Table 1; Figure 2).

The step width represents the horizontal distance measured between the position of the feet on the same event. Finally, foot progression angle can be measured, this represents the angle between the longitudinal axis of the foot and the line of gait progression (Table 1).

### 1.3. Kinetics Features

**Figure 3:**
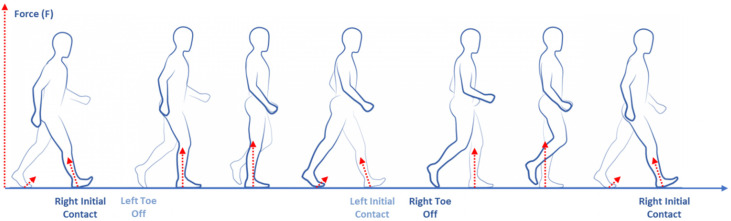
Gait kinetics (dynamics) features.

The kinetics analysis (or dynamics of gait) is the study of the forces and their effect on motion. The dynamics forces are the causes of the motion that result in the kinematic movements. Commonly, these forces are represented by the ground reaction force (GRF) on the hip, knee and ankle joints calculated on the sagittal plane. GRF is described only when feet are in contact with the ground (stance phase) and represents the effect of gravity on a body area counterbalanced by the contact with ground and the limb muscular activation [31,32] (Figure 3). GRF refers to a center of pressure (CoP) that is the point of force application.

### 1.4. Kinematics Features

**Figure 4:**
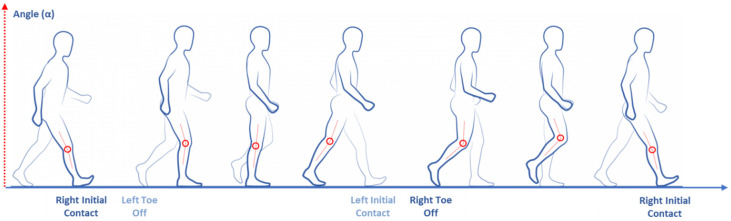
Gait kinematics features.

Kinematics features describe the movements without taking the forces causing the movements into account [31,32]. Kinematics analysis could describe gait features based on the sagittal, horizontal or frontal plane for several body areas and joints such as the ankle, knee, hip and pelvis. Kinematics features could be extrapolated both from the stance and swing phases. The kinematic analysis of the position, velocity and acceleration of a body part can be determined. Angular kinematics objectively quantify (as degrees) the joint’s motion around axes in different gait phases (Figure 4). 

### 1.5. Gait Analysis Technologies

Several technologies can be used for quantitative gait analysis. Technologies can be divided into two main subtypes: wearables and non-wearables [33]. Wearable sensors used for gait analysis are inertial sensors [34], goniometer [35], pressure and force sensors [36], electromyography (EMG) [37,38], IR-UWB (impulse radio ultra-wideband) [39] and ultrasound [40]. Among non-wearable sensors, the most common types are floor sensors [41,42] and image processing-based technologies (such as a single or multiple cameras [43,44,45], time of flight [46,47,48], stereoscopic vision [49,50], structured light [51] and IR thermography [52]).

**Figure 5:**
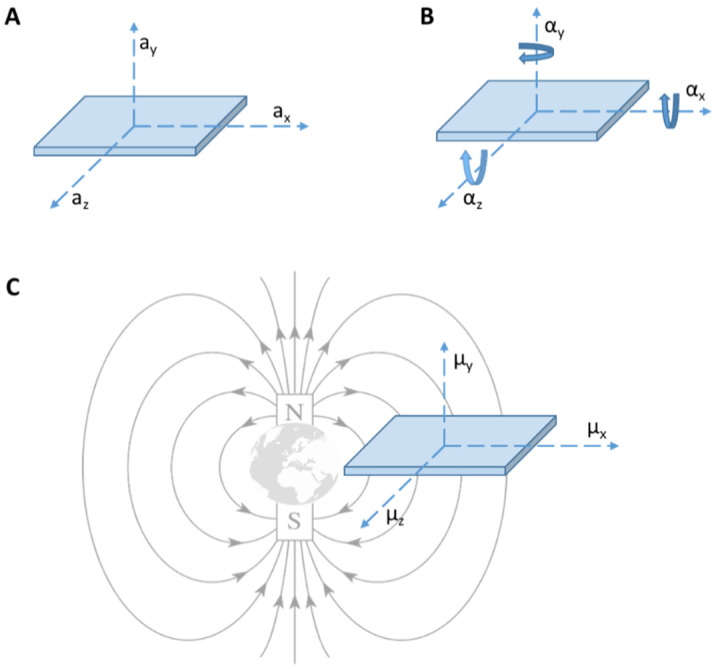
The same inertial measurement unit (IMU) device composed by accelerometers (**A**), a gyroscope (**B**) and a magnetometer (**C**). Legend: (**A**) a_x_, a_y_ and a_z_ = linear acceleration on the three axis x, y and z; (**B**) α_x_, α_y_ and α_z_ = angular acceleration on the three axis x, y and z and (**C**) μ_x_, μ_y_ and μ_z_ = magnetic moment on the three axis x, y and z.

Accelerometers, gyroscopes and magnetometers can be the component of the same inertial measurement unit (IMU) device (Figure 5), one of the most widely used type of sensors in gait analysis especially in PD [33,53]. It can measure velocity, acceleration, orientation and gravitational forces and can be used to study gait initiation [54], assess standing balance [55] and quantify bradykinesia [56]. 

Accelerometers are composed of a mechanical sensing element with a proof mass attached to a mechanical suspension system, with respect to a reference frame, that can be forced to deflect by the inertial force according to the acceleration of gravity. The acceleration can be measured electrically using the physical changes in the displacement of the proof mass with respect to the reference frame [34]. Accelerometer can be attached to the feet, legs or waist [33]. Gyroscope is based on the property that all body that revolves around an axis develop rotational inertia determined by the body’s moment of inertia [33]. Basically, a gyroscope is an angular velocity sensor. Magnetometer is based on the magneto resistive effect and can estimate changes in the orientation of a body segment in relation to the magnetic north. It can provide information that cannot be determined by both an accelerometer and gyroscope [34]. 

Goniometers work with resistance that changes depending on how flexed the sensors is. When flexed, the resistance increases proportionally to the flex angle. Goniometers are easy to set up and use a simple algorithm [33]. Goniometers are commonly used to study the angles for ankles, knees, hips and metatarsals [35].

Pressure and force sensors measure the force applied on the sensor without considering the components of this force on all other axes [33].

Force sensors measure the ground reaction force under the foot and return a current or voltage proportional to the pressure measured. Usually this kind of sensor is easily integrated into instrumented shoes [36]. Pressure and force sensors have been used to study stride length variability in PD patients with freezing of gait (FOG) [57]. Electromyography is a neurophysiologic technique that registers the electrical signals associated with motor unit activity, both voluntary and involuntary. The electrical signal can be recorded with surface electrodes (non-invasively) or needle electrodes (invasively) [58]. Some EMG electrodes are commercialized in combination with wireless technology and play an important role in evaluating walking performance during gait [38]. EMG can be used to study postural disorders in Parkinson’s disease patients, like exploring muscular activity in the Pisa syndrome [59].

The impulse radio ultra-wideband (IR-UWB) technique can detect and track movements non-invasively with high resolution and accuracy through emitting impulse radio waves of very short duration, and receiving the reflected waves from the target body [60]. It has been used to quantify activity measurement in movement disorders [61]. This technology also shows a good penetrating power able to detect the motions of internal organs. This technique can also be used for a wearable healthcare system to continuously estimate foot clearance due to its high temporal resolution, low power consumption and multipath immunity [62]. This technology can be used for step and gait phase detection [39].

Ultrasonic sensors can measure the time a sound takes to send and receive the wave produced as it is reflected from an object. Knowing the time and the speed, we can estimate the distance between two points [33]. This kind of technology is useful for step length measurement and gait phase detection [40] to analyze bilateral gait symmetry and coordination [63].

Among non-wearable sensors, the single camera image processing system is composed of single or multiple cameras that can be used to obtain information about gait in selected individuals. This technique allows individual recognition and segment position localization. Image processing has been used to identify people by the way they walk [44] and has several medical applications such as gait recognition considering changes in the subject path [64], and study of the gait kinematic [65].

Time of flight (TOF) systems are based on cameras using signal modulation that measure distances between the camera and the subject based on the phase-shift principle [46]. The TOF system can detect the segment position, gait phase, foot plantar pressure distribution and are useful for individual recognition [47]. TOF systems have been used to assess medication adherence in patients with movement disorders [66].

Stereoscopic vision is used to determine the depth of points in the scene by using a model through the calculation of similar triangles between the optical sensor, the light-emitter and the object in the scene. This could be useful in gait phase detection, segment position and individual recognition [49].

Structured light is the projection of a light pattern under geometric calibration on an object whose shape is to be recovered [33]. This technology is used for segment position study and gait phase detection. Kinetic sensor is one of the most common devices using this technology to create a marker-based real-time biofeedback system for gait retraining [51].

Infrared thermography (IR) creates visual images based on surface temperatures. For studying human gait, its functioning is based on skin emissivity. This method has been applied to recognize the human gait pattern [52].

### 1.6. Machine Learning Algorithms Application for Gait Analysis

There has been increasing use of machine learning (ML) in medicine including neurology to aid diagnosis, and patient management using risk stratification [67,68]. ML algorithms learn from data (past experiences) by identifying underlying patterns and relationships. The field of ML can broadly be categorized into supervised, unsupervised and reinforcement learning.

Supervised learning (SL) begins with the aim of predicting a known output or target. Indeed, an SL algorithm takes a known set of input data (the training set) and known responses to the data (output), and trains a model to generate reasonable predictions for the response to new input data. In such algorithms, the artificial intelligence (AI) is approximating what a trained physician is already able to perform with high accuracy. This approach means that the learning algorithm generalized the training data to previously unobserved situations in a “reasonable” way.

All forms of SL algorithms can be classified as either classification or regression. Classification techniques predict discrete responses. Regression techniques, instead, are used to predict continuous responses. They can also be used for modeling the risk, meaning that the computer is doing more than merely reproducing the physician skills. These algorithms are also capable of discovering new associations not apparently evident to human’s preliminary interpretation.

Differently from SL, in the unsupervised learning (UL) algorithm, we were no longer interested in predicting outputs. Instead, we aimed to discover naturally occurring patterns or groupings within the data. It is important to emphasize that the examples given to learners were unlabeled; thus, there was no error or reward signal to evaluate a potential solution. Common UL clustering algorithms could broadly divided into three groups: hard clustering, where each data point belongs to only one cluster, and soft clustering, where each data point can belong to more than one cluster; and dimensionality reduction techniques.

Reinforcement learning (RL) is an approach in ML that states what actions an agent should take in an environment to capitalize on the idea of an increasing reward. RL is different form standard SL in that correct input/output pairs are never presented, nor are suboptimal actions explicitly corrected. The primary goal is the direct performance, which involves finding a balance between exploration of unknown datasets and exploitation of current knowledge [69].

The most widely used ML technique in gait analysis is SL, with varying levels of complexity and interpretability. Table 2 describes the most used algorithms in this field.

## 2. Materials and Methods

In line with the study of Sánchez-Ferro, et al. [75] we used a similar search string for axial symptoms in Parkinson’s disease patients, in PubMed for articles published between 1 January 2005, and 30 August 2019 (Table 3). We identified studies that used technologies to distinguish PD patients from healthy subjects or to differentiate PD motor status or different disease stages. We only selected studies that declared a sensitivity and specificity of at least 80% when using gait analysis for either diagnosis or motor status discrimination. Additionally, further relevant articles based on the author’s knowledge of the state of the art in this field were also added.

For each selected study, we collected data about the technology, the algorithm used and its performance metrics like accuracy, sensitivity and specificity. In addition for studies, which declares only the regular accuracy ((true positives + true negatives)/(true positives + true negatives + false positives + false negatives)), the balanced accuracy ((sensitivity + specificity)/2) was calculated. This is because for unbalanced test sets, balanced accuracy is a better index for accuracy than regular accuracy [76].

## 3. Results

### 3.1. Discrimination of Parkinson’s Disease from Healthy Subjects

According to the inclusion and exclusion criteria, after the literature search and studies screening, 10 studies were selected that focused on distinguishing Parkinson’s disease patients from healthy subjects with gait analysis. To distinguish PD from healthy subjects, several technologies can be used (Table 4 and Table 5). One study used the data collected from wireless inertial sensors (Micro-attitude and heading reference system (AHRS) model, MicroStrain, Inc, Williston, VT, USA) placed on the foot in PD patients and healthy subjects to detect peculiar gait features and distinguish PD patients from controls [77]. In particular, authors detected physical kinematic features of pitch, roll and yaw rotations of the foot during walking and used principal component analysis (PCA) to select the best features that were subsequently used for the SVM method to classify PD patients, with and without gait impairment, and healthy subjects. From 67 collected features, they selected 15 kinematic features divided in three categories: pitch, roll and yaw features. The proposed classification has very high sensitivity, specificity and positive predict values (93.3%, 95.8% and 97.7% respectively) to distinguish PD patients from healthy subjects [77].

Another study compared two machine learning algorithms (decision-tree and neural networks) to differentiate healthy subjects gait patterns in different disease conditions in an elderly population (including patients affected by PD, hemiplegia, leg pain and back pain). Authors used movements data obtained from 12 retroreflective tags placed on the body captured by an infrared camera (Smart IR motion capture system) [78]. They studied 45 healthy controls and 25 PD patients. Predictors were based on velocity and calculated body distances (i.e., difference between average distance between right elbow and right hip and average distance between right wrist and left hip or the angle between two body segments). Global classification accuracy was high for both systems and reached over 95% for decision tree and more than 99% for neural network [78]. 

Moreover, Barth, et al. [79] demonstrated a good sensitivity and specificity (88% and 86% respectively) to differentiate healthy subjects and early PD patients using only a single mobile inertial sensor (gyroscope and accelerometer, integrated in the SHIMMER Company system) placed over the shoes. The patients performed standardized gait tests and from this data step, signal sequence and frequency features were extrapolated and used as predictors for the linear discriminant analysis (LDA). Moreover, they demonstrated that this system was able to distinguish between mild and severe gait pattern with high sensibility and specificity (100%).

Another approach was used by Yoneyama, et al. [80]: they used a single accelerometer placed on the waist and performed gait analysis to compare a continuous 24-h assessment of 10 PD patients and 17 healthy controls [80]. They used a gait detection algorithm based on the gait cycle (i.e., stride-to-stride time interval) and gait-induced acceleration relationship using 3 features of gait (high intensity, periodicity and biphasicity of gait) that introduced a set of indices in order to quantify subject’s walking mode and to assess daily gait characteristics. All the calculated indices were smaller in the PD group, and the proposed method was able to distinguish the PD gait from the normal gait with 100% sensitivity, 94.1% specificity and 96.3% accuracy. These results suggest that the afore-mentioned systems could differentiate normal subjects from those with movement disorders [80]. 

To easily and objectively assess the difference between PD patients and healthy subjects, Kugler, et al. [81] proposed a classification algorithm based on data collected from surface wireless EMG (Delsys Trigno, Delsys Inc., Boston, MA, USA) positioned on two inferior limbs muscles during the performance of standardized gait tests in five PD patients and five healthy subjects [81]. Furthermore, data from accelerometers (Trigno sensor) placed on heels were collected and used for step segmentation. Statistical and frequency features from EMG signals were used to train an SVM algorithm for step detection. The proposed step detection method reached 98.9% sensitivity and 99.3% specificity and the classification accuracy to distinguish between PD and healthy subjects reached 90% sensitivity and 90% specificity (average value).

Arora, et al. [82] investigated the feasibility and the accuracy of smartphones’ built-in tri-axial accelerometer (in LG Optimus S) developing an app to objectively assess PD patients and distinguish them from healthy subjects [82]. They studied 10 PD patients and 10 controls for 1 month and during execution of the gait tests to extract 23 features of frequency and time domain from accelerometer and subsequently used a random forest method to distinguish between PD and controls. This system reached a 98% balanced accuracy, 98.5% sensitivity and 97.6% specificity.

Another study proposed the use of a Bayesian gait recognition method based on data acquisition by video infrared camera system (Microsoft Kinect depth sensors) [83]. This system consisted of an infrared projector and two infrared cameras (on left and right) that follow the structured light principle. The collected data are converted in the depth frame matrix, depth frame contour, image frame matrix and skeleton numbering. Then the acquired data were further analyzed through MATLAB software. Eighteen PD patients, eighteen healthy subjects and fifteen healthy students were assessed and probabilistically classified according to their detected gait featured (stride length and gait speed) through skeletal tracking. This Bayesian system used the stride length, gait speed and age as features and was able to distinguish between PD patients and controls with 92.2% accuracy combining stride length and gait speed, and 94.1% accuracy combining stride length and patient/healthy subjects’ age.

Djurić-Jovičić, et al. [84] used an electronic walkaway to distinguish between the PD patient and controls. Authors compared 40 de novo PD patients and 40 controls while walking selecting three different tasks: normal pace walking, dual motor task (as walking and carrying a glass of water) and walking during mental task execution. The most relevant predictor variables were selected (19 features) including the stride length, stride length coefficient of variation (CV), swing time, step time asymmetry and heel-to-heel base support CV. These features were selected with the random forests algorithm and the classification accuracy of these selected features was tested with the support vector machine. The overall accuracy combining the three conditions was 85% to identify de novo PD patients from healthy subjects, with a sensitivity of 85% and a specificity of 82%. Their study also found that step time asymmetry and the support base CV are the most relevant factors that contribute to global system accuracy.

Alam, et al. [85] proposed a novel mathematical method to assess the gait in PD patients and controls using data from eight sensors below each foot and extrapolated features from vertical ground reaction force (VGRF) data recorded during subjects walking [85]. Twenty-nine PD patients and eighteen healthy subjects were enrolled in this study. Three different algorithms (sequential forward selection, minimum redundancy maximum relevancy feature selection (MRMR) and the mutual information-based feature ranking method) were applied to select the best features extracted from VGRF data and 13 features (like CV swing time, CV stride time and centre of pression data) were chosen. Finally, four different machine learning classifiers (SVM, k-nearest neighbor-kNN, random forest and decision trees) were compared to distinguish the gait pattern between healthy subjects and PD patients. The accuracy of the machine learning methods ranged from 85.21% (kNN) to 95.7% (SVM cubic kernel). SVM with cubic kernel showed a sensitivity of 94.4% and a specificity of 96.6%.

Finally, Pham and Yan [86] used a tensor decomposition algorithm called canonical polyadic decomposition (CPD) also known as Parallel Factor Analysis (PARAFAC), a generalization of PCA, to differentiate the multisensors time series of the gait between PD and controls in a previous published dataset of 93 PD patients and 72 controls [86]. Data were collected from load sensors (Ultraflex Computer Dyno Graphy, Infotronic Inc., Vriezenveen, NL, USA) placed on each shoe that recorded force in a function of time. Tensor-decomposition factors of control and PD patients showed a distinct relationship. This system used the full length of the VGRF time without considering the minimum number of strides required for effective tensor decomposition analysis of the gait dynamics. This system can be applied for very short time duration signals and can resolve the problem of obtaining several trials for stable and trustable results. Authors showed 100% of accuracy, sensitivity and specificity to distinguish PD and controls.

### 3.2. Parkinson’s Disease Motor Status Discrimination

According to the inclusion and exclusion criteria, after a literature search and studies screening, only three studies were selected and these papers aimed at identifying different motor statuses in Parkinson’s disease (Table 6 and Table 7). Sensors can be used to discriminate the different motor status in individual PD patients, in a single disease’s stage, monitoring the motor fluctuations, or can be used in a longitudinal way to monitor the motor status changes during the disease evolution.

Samà, et al. [87] focused their research on automatic detection of bradykinesia, the cardinal symptom of PD [87]. They proposed a mathematical algorithm to automatically identify bradykinesia in PD patients at home using an SVM classifier (that detects strides) based on data collected from a single accelerometer placed on the waist combined with a video-recording of the examination, they correlated the stride frequency with the UPDRS bradykinesia score. Their methods showed a high accuracy (>90%) to identify bradykinesia, a high sensitivity and specificity (92.52% and 89.07%, respectively) and a good correlation with UPDRS specific items.

To objectively detect the motor on–off fluctuations, an integrated system like REMPARK (personal health device for the remote and autonomous management of Parkinson’s disease, FP7 project REMPARK ICT-287677) was used [88]. The REMPARK system consists of an algorithm added in an app inside a smartphone that used the data from an accelerometer placed on the iliac crest. This system was developed for longitudinal evaluation. In this study, 41 PD patients were enrolled for a 3-day monitoring. For the on/off state discrimination, authors developed an algorithm, which analyzed gait [89] and dyskinesias [90]. The algorithm responses were compared to a self-reported on–off diary. The REMPARK system showed 97% sensitivity in detecting off states and 88% specificity in detecting on phases compared to diaries.

Barth, et al. [79] demonstrated, in a cohort of 14 early stage PD patients and 13 intermediate PD stage patients, that a mobile and light inertial sensor (gyroscope and accelerometer, integrated in the SHIMMER system) placed over the shoes allows differentiation between the two groups with 100% sensitivity and specificity with using a linear discriminant analysis (LDA) classifier that combines step, signal sequence and frequency features as predictors [79].

## 4. Discussion

Several studies aimed at detecting specific patterns of gait alterations in Parkinson’s disease by using a quantitative technology-based assessment [28,29,91,92]. Gait impairment is an evolving condition throughout the progression of the disease and different patterns of gait disturbances can be detected in early, mild to moderate and advanced stages [28]. 

Early specific alterations include reduced amplitude of arm swing, smoothness of locomotion and increased interlimb asymmetry [29]. Impaired muscle contraction, rigidity and postural instability contribute to reduced forward limb propulsion, which, in turn, can negatively affect spatiotemporal gait parameters, such as speed and step length [29]. In particular, reduced step length seems to be a specific feature of Parkinson’s disease gait [92]. Sensor-based observations showed that the increased variability in gait reflects increased gait instability that can be detected early in the disease and can be a useful marker of disease progression [91,93]. To carry out two tasks at the same time, a paradigm known as dual-task interference, is particularly complex in PD patients because of two independent effects influencing gait: the first is an age-associated reduction in gait performance unrelated to pathology, and the second one is a PD-specific effect due to a dual-task coordination deficit interfering with postural control. The latter suggests reduced stability and ability to adapt to PD patients under dual-task conditions [93]. Arm swing outcomes provide a sensitive measure of decline in gait function in PD under dual-task conditions [91]. On the other hand, one of the most representative early feature of Parkinsonian gait, reduced speed, is not disease specific [28].

In the mild-to-moderate stage, symptoms spread bilaterally so that asymmetry might decrease [94]. Gait problems worsen and shuffling steps, increased double-limb support and increased cadence become common [28]. Motor automaticity becomes further impaired, resulting in fragmented motor function, such as defragmentation of turns (i.e., turning en block) and problems with gait initiation [30].

Further worsening in gait characterizes the advanced stage of the disease, with more frequent freezing of gait (FOG) and motor blocks, reduced balance and postural control, motor fluctuations and dyskinesia [28]. 

The use of several sensors technologies to objectively assess Parkinson’s disease (PD) symptoms has exponentially increased in the last twenty years. Despite different features such as analysis of the face [95,96], speech [97,98], bradykinesia [15,16,17], rigidity [17,18,19,20] and tremor [21,22,23] being explored for objective PD evaluation, gait analysis has received widespread attention as part of an objective assessment for PD patient examination. Gait analysis is based on capturing movement with a motion capture device that can be wearable or non-wearable. The analysis of the acquired signals used different statistical or machine learning algorithms like the support vector machine (SVM), dynamic neural network (DNN), naïve Bayes, random forests or decision tree. For machine learning algorithms, the more complex the algorithm, the more likely it is able to determine an optimal decision boundary and hence improved accuracy. However, this improvement in accuracy comes at the cost of reduced interpretability. Among the algorithms included in this survey, decision trees are the most interpretable. All the algorithms used data derived from several features of the gait pattern, which can be grouped into three parameters groups: spatiotemporal, kinematic and kinetic [85]. Spatial parameters measure the physical distance between two steps (like strength length); temporal parameters evaluate the time spent to complete a gait cycle (like the cadence, duration of swing and stance phase) and kinematic parameters evaluate the movement of an object without consideration of its cause while kinetic parameters measure the force that cause the movement (like the ground reaction force during walking) [85].

The majority of these studies assessed if the motion capture device and associated algorithms can distinguish between PD patients and healthy subjects. Other studies investigated if these tools could help to classify different motor status of PD or identify various disease stages.

Among studies focused on discriminating PD vs. healthy subjects, various studies showed high accuracy (more than 90%; Table 4). In addition, regarding studies focused on motor status discrimination, Bayes, et al. [88] were able to discriminate the on/off state by merging an algorithm, which analyzed gait and dyskinesia; instead Barth, et al. [79] were able to differentiate the early vs. intermediate PD stage, with 100% sensitivity and specificity (Table 6). However, it should be considered that all these algorithms need to be validated on larger and representative populations in order to avoid overfitting the problem, which makes the algorithm valid only for the analyzed sample. In addition, the simplest algorithms that provide acceptable accuracy are preferable (i.e., logistic regression and decision trees) over more complex algorithms (e.g., SVM and neural networks) that may provide slightly higher accuracy but are less interpretable. 

## 5. Conclusions

The present overview showed that among the high volume of literature, published on the topic of objective gait analysis in PD, only few studies showed accurate algorithms that can potentially be clinically useful for diagnosis and symptoms monitoring. However, none of those studies have been independently validated or tested on a large scale.

## Figures and Tables

**Table 1:** Spatiotemporal gait and stride features.

**Gait Cycle**	The time from initial contact to initial contact on the same foot including both the stance phase and swing phase.
**Stance Phase**	The period during which the foot is in contact with the support surface during one gait cycle.
**Swing Phase**	The period during which the foot is airborne during one gait cycle.
**Double Limb Support**	The period during which both feet are in contact with the support surface during one gait cycle.
**Single Limb Support**	The period during which only one foot is in contact with the support surface during one gait cycle.
**Step Duration**	The period between 2 successive events of the same type on opposite limbs.
**Stride Length**	The linear distance between 2 successive events (initial contact) on the same limb.
**Step Length**	The linear distance between 2 successive events of same type on opposite limbs.
**Step Width**	The horizontal distance between 2 points on opposite limbs.
**Foot Progression Angle**	The angle between the longitudinal axis of the foot and the line of gait progression.

**Table 2:** Machine learning algorithm used for gait analysis.

Algorithm	How It Works	Interpretability (+): Min (+++++): Max
**k Nearest Neighbor (kNN):**	Categorizes objects based on the classes of the nearest neighbors in the dataset. The function is estimated only locally and all of the calculations are delayed up to the prediction or classification. The kNN method is sensitive to the dataset [70].	+++
**Linear Support Vector Machine (SVM):**	Classifies data by finding the linear decision boundary (hyperplane) that separates all data points of one class from those of the other class [71]. The best hyperplane for an SVM is the one with the largest margin between the two classes, when the data is linearly separable [72,73].	+++
**Kernel Support Vector Machine (Kernel SVM):**	Similar to SVM but additionally uses the “kernel trick” to transform the input data (not linearly separable) into a new feature space (linearly separable)	++
**Artificial Neural Networks (ANNs)**	Inspired by the connectivity of neurons in the human brain, a neural network consists of highly connected networks of neurons that relate the inputs to the desired outputs [74]. Each nonlinear function in the network can be used for the mapping from the training inputs to the training outputs.	+
**Naïve Bayes (NB)**	A naïve Bayes classifier assumes that the presence of a particular feature in a class is unrelated to the presence of any other feature and uses the Bayes theorem to determine the posterior probability	+++
**Linear Discriminant Analysis (LDA)**	It classifies data by finding linear combinations of features. Discriminant Analysis (DA) assumes that different classes generate data based on Gaussian distributions. The distributions parameters are used to calculate boundaries, which can be linear or quadratic functions.	++++
**Decision Tree (DT)**	It predicts responses to data by following the decisions in the tree-algorithm from the root (beginning) down to a leaf node. DTs can solve a classification problem by continuously dividing the input space to build a tree on which the nodes are as pure as possible and contain points of a single class. DTs are considered naïve algorithms; however, they have great performances in prediction and classification applications.	+++++
**Random forest**	An ensemble technique that uses a very large number of decision trees, often resulting in improved accuracy over DTs at the expense of reduced interpretability	+

**Table 3:** Search strategy.

Domain	Search String
Disease	(“Parkinsonian Disorders” OR “Parkinson disease” OR “Parkinson Disease, Secondary” OR “Basal Ganglia Diseases” OR “Parkinsonism” OR “Parkinson’s Disease”) AND
Technology	(“Technology” OR “Technologies” OR “Diagnostic Techniques, Neurological” OR “Assessment” OR “Patient Outcome Assessment” OR “Symptom Assessment” OR “Evaluation” OR “Diagnostic Self Evaluation” OR “Investigative Techniques” OR “Wireless Technology” OR “Remote Sensing Technology” OR “Biomedical Technology” OR “Technology Assessment, Biomedical” OR “Medical Informatics” OR “Cloud Computing” OR “Point of Care systems” OR “Biomedical Engineering” OR “Machine Learning” OR “Artificial Intelligence” OR “Kinesis” OR “Mobile Applications” OR “Cell Phones” OR “Smartphones” OR “Software” OR “Software Validation” OR “Platform” OR “Accelerometer” OR “Gyroscope” OR “Magnetometer” OR “Actigraph” OR “Wearable” OR “Device” OR “Big Data” OR “Sensor” OR “Internet of Things” OR “Closed-loop System” OR “Hybrid” OR “Home monitoring” OR “Quantitative” OR “Algorithm” OR “Telemetry” OR “Instrumented” OR “Virtual Reality”) AND
Axial symptoms	(“Gait” OR “Gait Disorders, Neurologic” OR “Posture” OR “Posture Balance” OR “Freezing of Gait” OR “Gait Disturbances” OR “Postural Instability” OR “Falls” OR “Fall”) AND
Time range	(“2005/01/01”[PDAT]: “2019/08/30”[PDAT])

**Table 4:** Parkinson’s disease vs. healthy subjects discrimination.

Ref	Algorithm	N. Features	N. Patients/Healthy	Regular Accuracy	Balanced Accuracy	Sensitivity (%)	Specificity (%)
[77]	SVM	15		NA	94.6%	93.3%	95.8%
[78]	Decision tree	13	25/45	95%	92.3%	88.8%	95.8%
[78]	Neural Network	13	25/45	99%	100.0%	100.0%	100.0%
[79]	LDA	12	27/16	NA	87.0%	88.0%	86.0%
[80]	NA	3	10/17	96.3	97.1%	100.0%	94.1%
[81]	SVM	8	5/5	NA	90.0%	90.0%	90.0%
[82]	Random forest	23	10/10	NA	98.1%	98.5%	97.6%
[83]	Bayesian probability	2	18/33	92.2%	93.3%	94.4%	92.2%
[83]	Bayesian probability	2	18/33	94.1%	94.2%	94.4%	93.9%
[84]	SVM	19	40/40	85.0%	83.5%	85.0%	82.0%
[85]	SVM	13	29/18	95.7%	95.5%	94.4%	96.6%
[85]	Random forest	13	29/18	89.4%	89.3%	88.9%	89.7%
[85]	kNN	13	29/18	85.1%	84.8%	83.3%	86.2%
[85]	Decision tree	13	29/18	87.2%	87.6%	88.9%	86.2%
[86]	Tensor decomposition	16	93/72	100.0%	100.0%	100.0%	100.0%

Abbreviations: CoP: center of pressure; CV: coefficient of variation; kNN: k-nearest neighbor, LDA: linear discriminant analysis; NA: not available; SVM: support vector machine; VGRF: vertical ground reaction force.

**Table 5:** Parkinson’s disease vs. healthy subjects discrimination features selected.

Ref	Algorithm	Features
[77]	SVM	**Pitch** - Pitch range of motion - Maximum angle of dorsiflexion - Maximum angle of plantar flexion - Plantar flexion SD - Single-step maximum of maximum angle of plantar flexion **Roll** - Roll range of motion - Maximum positive roll angle - Maximum negative roll angle **Yaw** - Yaw range of motion - Maximum positive yaw angle - Maximum negative yaw angle - Overall 3D SD - Maximum cadence **Additional** - Single-step maximum of maximum negative roll angle - Single-step minimum of maximum negative roll angle
[78]	- Decision tree - Neural Network	- Absolute difference between i) average distance between right elbow and right hip and ii) average distance between right wrist and left hip. - Average angle of the right elbow. - Quotient between maximal angle of the left knee and maximal angle of the right knee. - Difference between maximal and minimal angle of the right knee. - Difference between maximal and minimal height of the left shoulder. - Difference between maximal and minimal height of the right shoulder. - Quotient between i) difference between maximal and minimal height of left ankle and ii) maximal and minimal height of right ankle. - Absolute difference between i) difference between maximal and minimal speed (magnitude of velocity) of the left ankle and ii) difference between maximal and minimal speed of the right ankle. - Absolute difference between i) average distance between right shoulder and right elbow and ii) average distance between left shoulder and right wrist. - Average speed (magnitude of velocity) of the right wrist. - Frequency of angle of the right elbow passing average angle of the right elbow - Average angle between (i) vector between right shoulder and right hip and (ii) vector between right shoulder and right wrist. - Difference between average height of the right shoulder and average height of the left shoulder.
[79]	LDA	**Step features** - Step duration - Rise gradient of swing phase - Fall gradient of swing phase - Standard deviation of minima - Maxima minima difference **Signal sequence** - Variance - Integral - Entropy **Frequency analysis** - Dominant frequency - Energy ratio - Energy in band 0.5–3 - Energy in band 3–8
[80]	NA	- High intensity, - Periodicity, - Biphasicity
[81]	SVM	**EMG statistics** - Variance - Skewness - Kurtosis - RMS Energy **EMG frequency** - Dominant Frequency - Mean Frequency - Median Frequency - Total Power
[82]	Random forest	- Mean - Standard deviation - 25th percentile - 75th percentile - Inter-quartile range - Median - Mode - Data range (maximum – minimum) - Skewness - Kurtosis - Mean squared energy - Entropy - Cross-correlation between the acceleration in x and y axis - Mutual information between the acceleration in x and y axis - Cross-entropy between the acceleration in x and y axis - Extent of randomness in body motion - Instantaneous changes in energy due to body motion - Autoregression coefficient at time lag1 - Zero-crossing rate - Dominant frequency component - Radial distance - Polar angle - Azimuth angle
[83]	Bayesian probability	- Stride length, - Gait speed
[83]	Bayesian probability	- Stride length, - Age
[84]	SVM	- Step time - Step time asymmetry - Stance % of cycle - Swing time - Swing time CV - Stride time - Stride time CV - Stride time asymmetry - Single support time CV - Heel off on time - Heel off on std- deviation - Double support time - Double support time CV - Double support load % of cycle - Step length asymmetry - Stride length - Stride length CV - Heel-to-heel support base - Heel-to-heel support base CV
[85]	- SVM - Random forest - kNN - Decision tree	- CV of swing time - CV of stride time - Mean CoP of x-coordinate - Standard deviation CoP of x-coordinate - Mean CoP of y-coordinate - Standard deviation CoP of y-coordinate - Mean peak force at heel strike - Mean peak force at toe strike - Standard deviation of peak forces at heel strike - Standard deviation of peak forces at toe strike - Mean kurtosis - Mean skewness - Mean Peak power of VGRF signal
[86]	Tensor decomposition	- VGRF measurements from 8 sensors for the foot

**Table 6:** Parkinson’s disease motor status discrimination.

Ref	Algorithm	N. Features	N. Patients	Regular Accuracy	Balanced Accuracy	Sensitivity (%)	Specificity (%)
[79]	LDA	12	27	NA	100.00%	100.00%	100.00%
[87]	SVM	1	12	91.81%	90.80%	92.52%	89.07%
[88]	NA	NA	41	NA	92.50%	97.00%	88.00%

Abbreviations: LDA: linear discriminant analysis; NA: not available; SVM: support vector machine.

**Table 7:** Parkinson’s disease motor status discrimination features selected.

Ref	Algorithm	Features
[79]	LDA	**Step features** - Step duration - Rise gradient of swing phase - Fall gradient of swing phase - Standard deviation of minima - Maxima minima difference **Signal sequence** - Variance - Integral - Entropy **Frequency analysis** - Dominant frequency - Energy ratio - Energy in band 0.5–3 - Energy in band 3–8
[87]	SVM	- Motion fluency value
[88]	NA	NA
